# Characterization of Non‐Motor Fluctuations Using the Movement Disorder Society Non‐Motor Rating Scale

**DOI:** 10.1002/mdc3.13520

**Published:** 2022-08-05

**Authors:** Daniel Johannes van Wamelen, Silvia Rota, Anette Schrag, Alexandra Rizos, Pablo Martinez‐Martin, Daniel Weintraub, Kallol Ray Chaudhuri

**Affiliations:** ^1^ Division of Neuroscience, Department of Basic & Clinical Neuroscience, King's College London Institute of Psychiatry, Psychology & Neuroscience London United Kingdom; ^2^ Parkinson Foundation Centre of Excellence at King's College Hospital NHS Foundation Trust London United Kingdom; ^3^ Department of Neurology, Centre of Expertise for Parkinson & Movement Disorders, Donders Institute for Brain, Cognition and Behaviour Radboud University Medical Center Nijmegen The Netherlands; ^4^ Department of Clinical and Movement Neurosciences UCL Institute of Neurology, University College London London United Kingdom; ^5^ Center for Networked Biomedical Research in Neurodegenerative Diseases (CIBERNED) Carlos III Institute of Health Madrid Spain; ^6^ Department of Psychiatry and Neurology Perelman School of Medicine at the University of Pennsylvania Philadelphia PA USA; ^7^ Parkinson's Disease Research, Education and Clinical Center (PADRECC) Philadelphia Veterans Affairs Medical Center Philadelphia PA USA

**Keywords:** Parkinson's disease, non‐motor fluctuations, non‐motor symptoms, progression, fluctuations, wearable sensor

## Abstract

**Background:**

Non‐motor fluctuations (NMF) in people with Parkinson's disease (PwP) are clinically important yet understudied.

**Objective:**

To study NMF in PwP using both the Movement Disorder Society Non‐Motor Rating Scale (MDS‐NMS) NMF subscale and wearable sensors.

**Methods:**

We evaluated differences in overall burden of NMF and of specific NMF across disease durations: <2 years (n = 33), 2–5 years (n = 35), 5–10 years (n = 33), and > 10 years (n = 31). In addition, wearable triaxial sensor output was used as an exploratory outcome for early morning “off” periods.

**Results:**

Significant between‐group differences were observed for MDS‐NMS NMF total scores (*P* < 0.001), and specifically for depression, anxiety, fatigue and cognition, with both NMF prevalence and burden increasing in those with longer disease duration. Whereas only 9.1% with a short disease duration had NMF (none of whom had dyskinesia), in PwP with a disease duration of >10 years this was 71.0% (*P* < 0.001). From a motor perspective, dyskinesia severity increased evenly with increasing disease duration, while NMF scores in affected individuals showed an initial increase with largest differences between 2–5 years disease duration (*P* < 0.001), with plateauing afterwards. Finally, we observed that the most common NMF symptoms in patients with sensor‐confirmed early morning “off” periods were fluctuations in cognitive capabilities, restlessness, and excessive sweating.

**Conclusions:**

Non‐motor fluctuations prevalence in PwP increases with disease duration, but in a pattern different from motor fluctuations. Moreover, NMF can occur in PwP without dyskinesia, and in those with NMF the severity of NMF increases most during years 2–5 after diagnosis.

Parkinson's disease (PD) is a neurodegenerative disorder characterized by progressive extrapyramidal symptoms and a range of non‐motor symptoms (NMS), such as neuropsychiatric and autonomic symptoms, sleep disturbances and pain.^1^ Broadly most, but not all, NMS tend to increase in frequency and severity over the course of the disease, with a consequent greater impact on patients’ quality of life,^1^, ^2^ although the pattern of progression appears to differ from motor symptoms.^3^ Classically, it is assumed that non‐motor fluctuations (NMF) accompany motor fluctuations when the latter almost invariably starts after levodopa treatment of PD.^4^ More recent evidence, however, suggests that NMF may, in fact, already occur during early disease when motor fluctuations are not yet apparent.^5^

The most common clinical scenario in people with Parkinson's (PwP) is NMF accompanying motor fluctuations, although the pattern of NMF may vary.^5^, ^6^, ^7^, ^8^ Despite the frequent temporal co‐occurrence of NMF with motor fluctuations, the impact of NMF is considered to be considerable on its own and in some cases more detrimental to quality of life than motor fluctuations.^9^ Whilst motor fluctuations have been extensively described and divided into distinct phenomena (eg, wearing‐off periods and dyskinesias), NMF on the other hand are less clearly objectively defined, and due to the lack of specific validated tools remain poorly recognized. Moreover, their specific relationship with motor symptoms and disease progression remains understudied.^8^, ^10^ The recently published NMF subsection of the Movement Disorder Society Non‐Motor Rating Scale (MDS‐NMS),^5^, ^11^ as well as other NMF scales such as the NoMoFa scale,^12^, ^13^ allow specific interrogation of NMF in PD. The MDS‐NMS is the successor to the Non Motor Symptoms Scale (NMSS)^14^ and one of the main changes include the presence of this dedicated NMF subscale, which comprises eight questions on specific NMF regarding individual symptom severity and overall frequency.^11^ In this analysis we provide a more comprehensive overview of NMF, using this scale combined with wearable sensor data, and investigate their association with other symptoms in PD. Based on previous studies, including a study by Storch et al., ^10^ we hypothesized that NMF burden in PwP would increase with increasing disease duration. We also hypothesized that NMF may start before overt motor fluctuations are present.

## Methods

### Source of Data

Data used in the current analyses were from the dataset used for the validation of the MDS‐NMS and have been described before.^5^, ^11^ In the UK, this study was approved for adoption to the UK Clinical Research Network (UKCRN No. 18003) and patients and controls provided written informed consent prior to study procedures in accordance with the Declaration of Helsinki. For the current analyses, we used the data collected at King's College Hospital London, UK. Data used included: sex, age at disease onset, disease duration, Levodopa equivalent daily dose (LEDD), Hoehn and Yahr (HY) staging, Movement Disorder Society Unified Parkinson's Disease Rating Scale (MDS‐UPDRS), and MDS‐NMS scores.

For a subset of PwP who were included in the MDS‐NMS validation study, wearable sensor data was available through the Parkinson's Kinetigraph™ Registry (PKG Registry) study which is currently active at the Parkinson's Foundation Centre of Excellence at King's College Hospital London, UK. This registry obtained ethical approval from the London – Riverside Research Ethics committee (REC reference: 17/LO/1010). All patients gave written informed consent, and the study was conducted in line with the Declaration of Helsinki. For a total of 81 participants in the study a PKG reading within 4 months of the MDS‐NMS completion was available. The cut‐off of 4 months has been deployed in previous studies,^15^ and during this period participants did not have a change of dopaminergic medication or medication dose.

### Data Characteristics and Processing

The primary aim of the current study was to evaluate differences in overall burden of NMF, and of specific NMF, as measured by the NMF subscale of the MDS‐NMS, across different disease durations of PD. For this, we divided the patient cohort (n = 132) into four different groups, depending on disease duration based on previously published cut‐offs^3^: group 1: <2 years (n = 33), group 2: 2–5 years (n = 35), group 3: 5–10 years (n = 33), and group 4: >10 years (n = 31). In addition, we sought to compare the distribution of magnitude of change in each NMS across the groups with different disease durations. For this, across the abovementioned four groups, we compared the distribution of each of the five severity levels of NMF (0: no change; 1: minimal; 2: small; 3: medium; and 4: large), as well the MDS‐UPDRS part IV scores for time spent with dyskinesia (0: normal; 1: slight; 2: mild; 3: moderate; and 4: severe). The Parkinson's Kinetigraph™ (PKG) used in this study is a wrist‐worn wearable device providing continuous monitoring of motor symptoms in PD over a period of six consecutive days, after which data is downloaded and analyzed using a proprietary algorithm to calculate scores for bradykinesia (BKS) and dyskinesia (DKS).^16^ Both scores are the median value of bradykinesia and dyskinesia, respectively, over a specific period during the day. These values have been shown to correlate with Unified Parkinson's Disease Rating Scale part III and modified Abnormal Involuntary Movement Scale scores assessed at the time of the wearable monitoring.^17^, ^18^ In addition, both BKS and DKS scores were available as scores over five three‐hourly epochs for the daytime (06:00–21:00) as follows: epoch 1) 06:00–09:00; epoch 2) 09:00–12:00; epoch 3) 12:00–15:00; epoch 4) 15:00–18:00; and epoch 5) 18:00–21:00. For each of these epochs, the percentage of time spent in severe bradykinesia or dyskinesia, defined as BKS or DKS values over the 75th percentile (BKS ≥ grade III and DKS ≥ grade III, respectively), was used for analysis as they are of functional significance and have been previously validated for us in PwP.^16^ We included PKG data if a participant had a PKG recording within 120 days of the MDS‐NMS assessment, based on previously used cut‐offs.^15^

For the wearable sensor outcomes, we compared: (a) dyskinesia severity scores across different disease durations in PD, in line with the MDS‐NMS analysis outlined above; (b) differences in recording dyskinesia severity between the wearable sensor and classical motor scales (MDS‐UPDRS); and (c) NMF severity in participants with and without early morning “off” periods (EMO) as a marker of motor fluctuations. The first epoch (06:00–09:00) was not used for dyskinesia analysis as some participants may still have been asleep during this time potentially providing unreliable readings.^19^ In order to achieve this, we compared the average dyskinesia severity over an entire day across four disease durations: group 1: <2 years (n = 16), group 2: 2–5 years (n = 21), group 3: 5–10 years (n = 22), and group 4: >10 years (n = 22). In order to assess the second aim (b) we described each individual NMF captured by the MDS‐NMS as either absent (score of 0 on the specific NMF in the MDS‐NMS NMF subscale) or present (score of 1 or over) and compared both the average complications and dyskinesia score obtained through the wearable sensor and dyskinesia scores assessed with the MDS‐UPDRS part IV total subscores (sum of questions 4.1–4.6) and dyskinesia scores (sum score of questions 4.1. and 4.2). Finally, for objective (c) we defined EMO as the presence of at least 5% higher absolute BKS75 scores in the 06:00–09:00 epoch compared to the 09:00–12:00 and based on this definition divided the participants into two groups: (1) without EMO, and (2) with EMO.

### Statistical Analyses

To assess the primary aim, group comparisons were made using Kruskal‐Wallis test. For comparison of the distribution of each of the five severity levels of NMF across the four different disease durations we used Chi‐Square test. With the wearable sensor data, group comparisons were made using Kruskal‐Wallis test. Group differences in NMF total scores between participants with and without EMO were compared through Mann–Witney *U* test for total NMF scores, and the degree of change for each of the eight separate NMF were compared using Chi‐Square test. For all correlations, we used Spearman's test.

For all analyses, a *P*‐value ≤0.05 was considered statistically significant, with a correction for multiple testing using Benjamini‐Hochberg procedure, where relevant. Data are presented as mean ± standard deviation, median (interquartile range) or number (percentage), unless otherwise specified. All data were analyzed using SPSS Version 27 (IBM SPSS Statistics for Windows, Version 27.0. Armonk, NY: IBM Corp.)

### Data Sharing

The data that support the findings of this study are available from the corresponding author upon reasonable request. Restrictions apply to the availability of these data, which were used for this study. Data are available only with the permission of the Movement Disorders Society.

## Results

### Non‐Motor Fluctuations

A total of 132 participants with PD were recruited to the MDS‐NMS validation study at King's College Hospital and were included in the current analyses. For 81 of the participants, wearable sensor data was available; the majority of participants had the wearable sensor recording within 30 days of the clinical MDS‐NMS assessment (74.1%). Demographics are provided in Table 1. No differences were observed between the group who had a PKG recording and the group who did not in relation to age (*P* = 0.655), disease duration (*P* = 0.120), LEDD (*P* = 0.104), MDS‐NMS scores (*P* = 0.379), MDS‐NMS NMF scores (*P* = 0.645), or MDS‐UPDRS part III and IV scores (*P* = 0.751 and *P* = 0.488).

**TABLE 1:** Baseline demographic and clinical characteristics in Parkinson's disease by disease duration

	Group 1: <2 years (n = 33)	Group 2: 2–5 years (n = 35)	Group 3: 5–10 years (n = 33)	Group 4: >10 years (n = 31)	Entire cohort (n = 132)	*P*	P*
Age (years)	61.58 ± 12.79	60.71 ± 12.25	63.91 ± 9.29	68.90 ± 10.82	63.65 ± 11.69	0.026	0.095
Disease duration (years)	1.18 ± 0.73	4.14 ± 0.88	7.42 ± 1.35	14.68 ± 3.47	6.70 ± 5.30	N/A	N/A
Sex (M/F)	17/16	21/14	19/14	20/11	77/55	0.761	0.797
LEDD (mg)	322.42 ± 432.63	621.25 ± 361.07	1480.91 ± 2073.19	1702.68 ± 1591.62	1015.43 ± 1427.74	<0.001	**<0.001** ^**a,b,c,d,e**^
Hoehn and Yahr stage	2.0 (1.5–2.5)	2.0 (2.0–3.0)	3.0 (2.0–4.0)	4.0 (3.0–4.0)	2.5 (2.0–4.0)	<0.001	**0.002** ^**a,b,c,e**^
MoCA	26.55 ± 4.42	26.86 ± 3.50	27.12 ± 3.24	24.90 ± 4.01	26.39 ± 3.86	0.039	0.107
MDS‐NMS – total score	46.88 ± 45.73	74.06 ± 71.96	71.97 ± 56.69	70.71 ± 46.64	65.69 ± 57.11	0.089	0.178
MDS‐UPDRS part I	8.91 ± 4.82	11.77 ± 7.84	11.30 ± 6.65	13.29 ± 5.57	11.30 ± 6.48	0.039	0.107
MDS‐UPDRS part II	7.67 ± 5.92	11.11 ± 8.48	12.45 ± 8.36	16.45 ± 8.21	11.84 ± 8.33	<0.001	**0.002** ^**a,e**^
MDS‐UPDRS part III	22.52 ± 17.02	26.06 ± 16.08	26.18 ± 16.02	31.45 ± 17.94	26.47 ± 16.86	0.188	0.295

Bold values indicate a *p*‐value of 0.05. Group‐wise comparisons *P* < 0.05: ^a^group 1–group 2; ^b^group 1–group 3; ^c^group 1–group 4; ^d^group 2–group 3; ^e^not all participants had an available wearable sensor recording (n = 16 for group 1, n = 21 for group 2, n = 22 for group 3, and n = 22 for group 4). *corrected for multiple testing using Benjamini‐Hochberg procedure. Data presented as mean ± standard deviation, number, or median (25th–75th percentile).

Abbreviations: M: male; F: female; LEDD: Levodopa Daily Equivalent Dose; MDS‐NMS: Movement Disorder Society Non Motor Scale; MDS‐UPDRS: Movement Disorder Society Unified Parkinson's Disease Rating Scale.

**FIG. 1:**
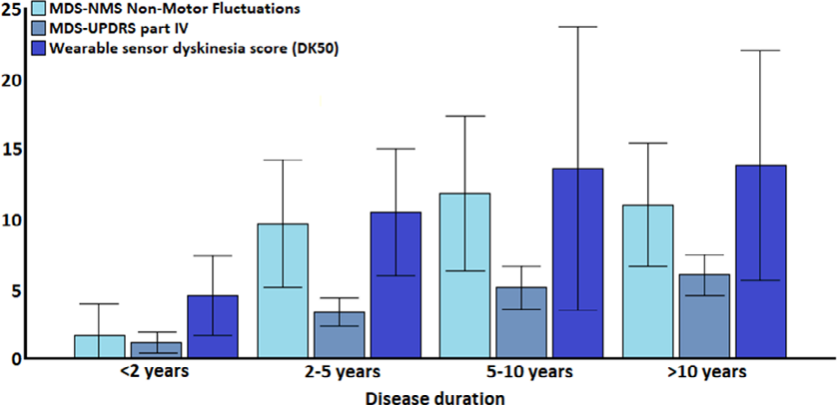
Distribution of non‐motor and motor fluctuation scores across different disease durations in people with Parkinson's disease. Abbreviations: MDS‐UPDRS, Movement Disorder Society unified Parkinson's disease rating scale; MDS‐NMS, Movement Disorder Society non‐motor scale; DK50, Median dyskinesia score. Both MDS‐NMS and MDS‐UPDRS scores worsened significantly (both *P* < 0.001), whereas a trend towards a statistically significant difference was observed for dyskinesia scores measured through a wearable sensor (*P* = 0.055). Values are expressed as mean and bars represent 95% confidence intervals.

Whereas only 9.1% with a short disease duration had NMF, in PwP with a disease duration of >10 years this was 71.0% (*P* < 0.001; Table 2). In PwP with NMF, NMF total scores were higher in those with longer disease duration (*P* < 0.001). In addition, higher dyskinesia and MDS‐UPDRS part IV total scores were observed in those with a longer disease duration (*P* < 0.001) (Table 2; Fig. 1). Nonetheless, the increase over time appeared different between NMF and MDS‐NMS part IV and dyskinesia scores, as the increase in both MDS‐NMS part IV and dyskinesia scores appeared steady with increasing disease duration, whereas the largest increase in NMF scores was during years 2–5 of disease duration; after this point NMF scores appeared to stabilize (Table 2; Fig. 1).

**TABLE 2:** Distribution of MDS‐NMS scores non‐motor fluctuation scores and motor fluctuations in people with Parkinson's disease

	Group 1: <2 years (n = 33)	Group 2: 2–5 years (n = 35)	Group 3: 5–10 years (n = 33)	Group 4: >10 years (n = 31)	Entire cohort (n = 132)	*P*	P*
Presence of any NMF	3 (9.1%)	19 (54.3%)	21 (63.6%)	22 (71.0%)	65 (49.2%)	<0.001	**<0.001**
MDS‐NMS non‐motor fluctuation total score	1.61 ± 6.31	9.60 ± 13.27	11.75 ± 15.30	10.94 ± 11.98	8.43 ± 12.70	<0.001	**<0.001** ^**a,b,c**^
MDS‐UPDRS total part IV	1.00 ± 2.14	3.23 ± 2.90	4.94 ± 4.37	5.84 ± 3.98	3.71 ± 3.87	<0.001	**<0.001** ^**a,b,c**^
MDS‐UPDRS dyskinesia^+^	0.00 ± 0.00	0.40 ± 0.88	0.82 ± 1.74	1.39 ± 1.87	0.64 ± 1.42	<0.001	**<0.001** ^**b,c**^
Wearable sensor dyskinesia score (PKG DK75)	1.2 ± 1.9	2.6 ± 2.7	3.0 ± 3.9	4.8 ± 7.4	3.0 ± 4.7	0.055	0.055^d^

Bold values indicate a *p*‐value of 0.05. ^+^Sum score of questions 4.1 and 4.2; Group‐wise comparisons *P* < 0.05: ^a^Group 1–group 2; ^b^group 1–group 3; ^c^group 1–group 4; ^d^not all participants had an available wearable sensor recording (n = 16 for group 1, n = 21 for group 2, n = 22 for group 3, and n = 22 for group 4). *Corrected for multiple testing using Benjamini‐Hochberg procedure.

Abbreviations: NMF, non‐motor fluctuations; MDS‐NMS, Movement Disorder Society Non‐Motor Rating Scale; MDS‐UPDRS, Movement Disorder Society Unified Parkinson's Disease Rating Scale; PKG, Parkinson's Kinetigraph™ (not available for all participants).

We also observed differences across disease duration groups in the frequency of individual NMF (Table 3). Specifically, we observed differences in NMF of depression, anxiety, cognitive abilities, and fatigue (*P* ≤ 0.040), with the >10 years duration group having the highest number of PD participants experiencing these NMF. Moreover, the daily time spent in non‐motor “off” state was higher in the groups with longer disease duration, with only 29.0% of PD participants with a disease duration of over 10 years having <10% non‐motor “off” time (*P* = 0.005; Table 3). At the same time, whereas in the group with a disease duration of 2 years or less no patient spent time with dyskinesia, 54.8% in the group with a disease duration of 10 years or more spent no time in dyskinesia as assessed on question 4.1. of the MDS‐UPDRS (*P* = 0.034; Table 3).

**TABLE 3:** Prevalence of non‐motor and motor fluctuation magnitudes of change across different disease durations in people with Parkinson's disease

Non motor scale non motor fluctuation subsection	Group 1: <2 years (n = 33)	Group 2: 2–5 years (n = 35)	Group 3: 5–10 years (n = 33)	Group 4: >10 years (n = 31)	*P*	*P* *
*NMF1 – depression*						
0 – No change	30 (90.9%)	22 (62.9%)	22 (66.7%)	17 (54.8%)	0.017	**0.031**
1 – Minimal	1 (3.0%)	2 (5.7%)	4 (12.1%)	7 (22.6%)		
2 – Small	0 (0.0%)	4 (11.4%)	5 (15.2%)	5 (16.1%)		
3 – Medium	2 (6.1%)	4 (11.4%)	2 (6.0%)	2 (6.5%)		
4 – Large	0 (0.0%)	3 (8.6%)	0 (0.0%)	0 (0.0%)		
*NMF2 – anxiety*						
0 – No change	31 (93.9%)	22 (62.9%)	19 (57.6%)	14 (45.2%)	0.008	**0.022**
1 – Minimal	0 (0.0%)	2 (5.7%)	4 (12.1%)	8 (25.8%)		
2 – Small	0 (0.0%)	5 (14.3%)	6 (18.2%)	5 (16.1%)		
3 – Medium	2 (6.1%)	3 (8.6%)	3 (9.1%)	3 (9.7%)		
4 – Large	0 (0.0%)	3 (8.6%)	1 (3.0%)	1 (3.2%)		
*NMF3 – thinking or cognitive abilities*						
0 – No change	33 (100.0%)	23 (65.7%)	20 (60.6%)	17 (54.8%)	<0.001	**<0.001**
1 – Minimal	0 (0.0%)	4 (11.4%)	2 (6.0%)	2 (6.5%)		
2 – Small	0 (0.0%)	6 (17.1%)	5 (15.2%)	11 (35.5%)		
3 – Medium	0 (0.0%)	1 (2.9%)	6 (18.2%)	1 (3.2%)		
4 – Large	0 (0.0%)	1 (2.9%)	0 (0.0%)	0 (0.0%)		
*NMF4 – bladder symptoms*						
0 – No change	32 (97.0%)	31 (88.6%)	26 (78.8%)	21 (67.7%)	0.065	0.089
1 – Minimal	0 (0.0%)	1 (2.9%)	1 (3.0%)	3 (9.7%)		
2 – Small	0 (0.0%)	2 (5.7%)	5 (15.2%)	7 (22.6%)		
3 – Medium	1 (3.0%)	1 (2.9%)	1 (3.0%)	0 (0.0%)		
4 – Large	0 (0.0%)	0 (0.0%)	0 (0.0%)	0 (0.0%)		
*NMF5 – restlessness*						
0 – No change	33 (100.0%)	28 (80.0%)	23 (69.7%)	23 (69.7%)	0.075	0.092
1 – Minimal	0 (0.0%)	1 (2.9%)	1 (3.0%)	1 (3.2%)		
2 – Small	0 (0.0%)	5 (14.3%)	7 (21.2%)	5 (16.1%)		
3 – Medium	0 (0.0%)	0 (0.0%)	0 (0.0%)	2 (6.5%)		
4 – Large	0 (0.0%)	1 (2.9%)	2 (6.0%)	0 (0.0%)		
*NMF6 – pain*						
0 – No change	31 (93.9%)	26 (74.3%)	22 (66.7%)	22 (71.0%)	0.389	0.389
1 – Minimal	0 (0.0%)	4 (11.4%)	2 (6.0%)	3 (9.7%)		
2 – Small	1 (3.0%)	2 (5.7%)	6 (18.2%)	4 (12.9%)		
3 – Medium	1 (3.0%	2 (5.7%)	2 (6.0%)	2 (6.5%)		
4 – Large	0 (0.0%)	1 (2.9%)	1 (3.0%)	0 (0.0%)		
*NMF7 – fatigue*						
0 – No change	31 (93.9%)	19 (54.3%)	19 (57.6%)	16 (51.6%)	0.022	**0.035**
1 – Minimal	1 (3.0%)	5 (14.3%)	3 (9.1%)	4 (12.9%)		
2 – Small	0 (0.0%)	6 (17.1%)	6 (18.2%)	7 (22.6%)		
3 – Medium	1 (3.0%)	4 (11.4%)	2 (6.0%)	4 (12.9%)		
4 – Large	0 (0.0%)	1 (2.9%)	3 (9.1%)	0 (0.0%)		
*NMF8 – excessive sweating*						
0 – No change	32 (97.0%)	35 (100.0%)	26 (78.8%)	26 (83.9%)	0.194	0.213
1 – Minimal	0 (0.0%)	0 (0.0%)	1 (3.0%)	0 (0.0%)		
2 – Small	1 (3.0%)	0 (0.0%)	2 (6.0%)	2 (6.5%)		
3 – Medium	0 (0.0%)	0 (0.0%)	3 (9.1%)	3 (9.7%)		
4 – Large	0 (0.0%)	0 (0.0%)	1 (3.0%)	0 (0.0%)		
*Time spent in non‐motor “Off” state*						
1 – Rarely (≤10% of waking day)	30 (90.9%)	16 (45.7%)	11 (34.3%)	9 (29.0%)	0.001	**0.004**
2 – Sometimes (11–25% of waking day)	1 (3.0%)	5 (14.3%)	6 (18.8%)	7 (22.6%)		
3 – Frequently (26–50% of waking day)	2 (6.1%)	12 (34.3%)	11 (34.3%)	13 (41.9%)		
4 – Majority of time (≥51% of waking day)	0 (0.0%)	2 (5.7%)	4 (12.5%)	2 (6.5%)		
*MDS‐UPDRS part IV – time spent with dyskinesia (item 4.1)*						
0 – Normal	33 (100.0%)	29 (82.9%)	26 (78.8%)	17 (54.8%)	0.015	**0.031**
1 – Slight	0 (0.0%)	5 (14.3%)	2 (6.1%)	8 (25.8%)		
2 – Mild	0 (0.0%)	1 (2.9%)	3 (9.1%)	3 (9.7%)		
3 – Moderate	0 (0.0%)	0 (0.0%)	1 (3.0%)	1 (3.2%)		
4 – Severe	0 (0.0%)	0 (0.0%)	1 (3.0%)	2 (6.5%)		
*MDS‐UPDRS part IV – time spent in motor Off (item 4.3)*						
0 – Normal	27 (81.8%)	13 (37.1%)	11 (33.3%)	6 (19.4%)	<0.001	**<0.001**
1 – Slight	3 (9.1%)	18 (51.4%)	9 (27.3%)	15 (48.4%)		
2 – Mild	3 (9.1%)	3 (8.6%)	11 (33.3%)	10 (32.3%)		
3 – Moderate	0 (0.0%)	1 (2.9%)	2 (6.1%)	0 (0.0%)		
4 – Severe	0 (0.0%)	0 (0.0%)	0 (0.0%)	0 (0.0%)		

^a^ Chi‐square test corrected for multiple testing using Benjamini‐Hochberg procedure.

Abbreviations: NMF, non motor fluctuation; MDS‐UPDRS, movement disorders society unified Parkinson's disease rating scale.

Bold values indicate a *p*‐value of 0.05.

We observed a moderate correlation between MDS‐NMS NMF scores and MDS‐UPDRS part IV scores (ρ = 0.657; *P* < 0.001).

### Wearable Sensor Outcomes

For 81 PwP wearable sensor data was available (17 PwP in group 1; 21 in group 2; 22 in group 3; and 24 in group 4). We observed a trend towards a statistically significant increase in average dyskinesia scores measured through a wearable sensor over time, with higher scores in PwP with a longer disease duration (*P* = 0.055; Table 2; Fig. 1). Secondly, we identified that particular NMF symptoms (ie, cognitive changes, restlessness, and excessive sweating) were more prevalent in participants with EMO (*P* ≤ 0.036; Table 4). In addition, NMF total scores were also higher in those with EMO (*P* = 0.028), despite the lack of between‐group differences in age, disease duration, and LEDD which are usually associated with EMO (Table 4).

**TABLE 4:** Non‐motor fluctuations in participants with Parkinson's disease with and without EMO

	No EMO (n = 36)	EMO (n = 45)	*P*	*P* *
Age (years)	64.14 ± 11.92	64.47 ± 10.03	0.433^a^	0.480^a^
Sex (M/F)	25/11 (69.4/30.6%)	18/27 (40.0/60.0%)	0.008^b^	**0.028** ^b^
Disease duration	6.61 ± 6.04	8.16 ± 5.06	0.066^a^	0.115^a^
Hoehn and Yahr stage	2.0 [2.0–4.0]	3.0 [2.0–4.0]	0.627^b^	0.627^b^
LEDD (milligram)	1092.61 ± 1597.38	1390.85 ± 1825.74	0.110^a^	0.154^a^
NMF total	3.69 ± 6.48	12.64 ± 15.73	0.008^a^	**0.028** ^a^
NMF1 – depression	9 (25.0%)	19 (42.2%)	0.105^b^	0.154^b^
NMF2 – anxiety	8 (/22.2%)	20 (44.4%)	0.037^b^	0.086^b^
NMF3 – cognition	6 (16.7%)	20 (44.4%)	0.008^b^	**0.028** ^b^
NMF4 – bladder symptoms	2 (5.6%)	9 (20.0%)	0.059^b^	0.116^b^
NMF5 – restlessness	2 (5.6%)	12 (26.7%)	0.013^b^	**0.036** ^b^
NMF6 – pain	7 (19.4%)	12 (26.7%)	0.446^b^	0.480^b^
NMF7 – fatigue	7 (19.4%)	25 (55.6%)	0.001^b^	**0.014** ^b^
NMF8 – excessive sweating	2 (5.6%)	7 (15.6%)	0.155^b^	0.197^b^

Bold values indicate a *p*‐value of 0.05. EMO was defined as at least 5% higher bradykinesia grade III (severe bradykinesia; 75th percentile bradykinesia and over) scores measured through a wearable sensor during the 06:00–09:00 epoch compared to the 09:00–12:00 epoch.

^a^ Mann–Whitney *U* test.

^b^ Chi‐Square test.

* Corrected for multiple testing using Benjamini‐Hochberg procedure.

Abbreviations: EMO, early morning off; M, male; F, female; LEDD, levodopa daily equivalent dose; NMF, non‐motor fluctuation.

We observed weak associations between wearable sensor dyskinesia scores and MDS‐NMS NMF scores (ρ = 0.392, *P* < 0.001), and MDS‐UPDRS part IV scores (ρ = 0.379, *P* < 0.001).

## Discussion

The key findings in the current study are that in PwP the prevalence and severity of NMF increases over the course of the disease and that in 9.1% of PwP NMF already occurred in the absence of dyskinesia. In PwP with NMF, the increase in severity was moreover not linear, but instead a sharp increase was observed following the first 2 years after diagnosis with plateauing of the severity thereafter. This progression pattern seemed to follow that of NMS burden in general. At the same time motor fluctuations showed a more consistent increase in severity. Moreover, even though the amount of time spent in non‐motor Off increased over time, not all NMF seemed to increase over this period, with particularly bladder symptoms, pain, and hyperhidrosis appearing to have a stable prevalence. Finally, we showed that NMF in cognitive capabilities, restlessness, and excessive sweating were more prevalent in PwP EMO, and that PwP with EMO had higher NMF total scores.

In line with previous studies, we observed that NMF burden increases with increasing disease duration. Nonetheless, the observed increase in burden appeared to be uneven, which is different from that observed in some previous studies. For example, Storch et al. recently showed that NMF severity increased with Hoehn and Yahr stage, but also with decreasing fluctuation amplitudes (differences in symptom severity between On and Off states) for both motor and NMF.^10^ Another study, however, showed that NMF are heterogeneous and complex, and that motor function did not correlate NMF severity.^20^ Moreover, Ossig et al. demonstrated low concordance rates between NMF and motor On–Off state.^21^ The discrepancies might be at least partially explained by using HY staging instead of disease duration and the use of different scales to assess NMF. Moreover, in our cohort we observed that also MDS‐NMS total scores increase with a similar pattern as NMF, ie, the largest increase occurred between the participants with a disease duration of <2 years and those with 2–5 years disease duration, largely in line with previous studies on NMS burden progression.^3^

The plateauing of NMF prevalence and severity in participants with longer disease duration in our analysis could perhaps be explained by an increase in the severity of non‐motor symptoms during ON periods in the more advanced stages, with a consequent reduction in the magnitude of symptom changes among On and Off states, coupled with a more severe motor phenotype as has been suggested by previous authors.^10^, ^22^ However, we noticed that the proportion of participants reporting larger magnitude of changes in specific NMS, particularly in relation to depressive symptoms and cognitive abilities, increased with increasing disease duration. This would suggest that the aforementioned suggestion does not satisfactorily explain the observed plateauing. Instead, it would seem that the overall perception of NMF burden seems to decrease over time in PwP. From previous studies the additional burden, on top of non‐motor symptoms alone, placed by NMF on quality of life remains largely unclear,^9^ and it might be speculated that some non‐motor symptoms become more continuous with more advanced disease stages.

Our study is among the first to examine the association of specific NMF with the occurrence of EMO in PwP, which we measured using a novel approach by using wearable sensor data to define EMO. We found that those with EMO had higher occurrence of NMF, which reached statistical significance for fluctuations in cognition, restlessness and fatigue. Whilst we did not measure NMF during the occurrence of EMO, our findings are in line with Rizos et al. who showed that 88.0% of PwP with EMO had worsening of NMS during the early morning period, mostly with an increase in urinary urgency and anxiety, but also dribbling of saliva, pain, low mood, limb paraesthesia and dizziness.^23^ The area of NMF during EMO should be explored further as the prevention of EMO by adjusting or optimizing dopaminergic treatment might improve these symptoms which tend to have a clear impact on quality of life.^24^, ^25^

Some limitations to our study need to be acknowledged, foremost the fact that our analyses were performed on a cross‐sectional cohort of participants and further studies with a long‐term longitudinal design to examine the evaluation of NMF should be performed. In addition, we used wearable sensor outcomes that did not necessarily coincide with the assessment of NMF, and some data was obtained up to 4 months from NMF assessment, reflecting clinical practice of use of wearables and in keeping with other studies.^15^ In addition, we only observed weak associations between wearable sensor data and MDS‐UPDRS part IV scores which might be perceived as limiting the use of wearable sensor data. However, the lack of strong association between scale‐based assessments and objective data has been reported before and highlights the limitations and bias related to scale‐based assessments.^26^, ^27^ In addition, sensors may record and interpret physiological movement patterns as dyskinesia. Overall, we feel that the relatively large sample size and the structured and consistent assessment of NMF provides novel data on the occurrence and development of NMS with advancing disease severity in patients with PD.

In summary, by using the novel NMF subscale of the MDS‐NMS we showed that NMF prevalence and severity increases with disease duration in a non‐linear pattern with the largest increase in severity occurring after the first 2 years after PD diagnosis, mirroring NMS burden progression. Moreover, NMF can occur in PwP without dyskinesia showing that the two are not necessarily linked. This analysis also underlines the feasibility of the MDS‐NMS scale with its dedicated NMF subscale in detecting NMF. In addition, we showed that this increase in NMF was largely driven by fluctuations in bladder symptoms, pain, and hyperhidrosis NMF increasing over time, with stable prevalence of other NMF. Further research is needed into the factors determining the progression of NMF and how impact on quality of life might chance over time.

## Author Roles

(1) Research Project: A. Conception, B. Organization, C. Execution; (2) Statistical Analysis: A. Design, B. Execution, C. Review and Critique; (3) Manuscript: A. Writing of the first draft, B. Review and Critique.

DvW: 1A, 1B, 1C, 2A, 2B, 2C, 3A, 3B.

SR: 1B, 1C, 2C, 3A, 3B.

AS: 2C, 3A, 3B.

AR: 2C, 3A, 3B.

PMM: 1A, 1C, 2C, 3A, 3B.

DW: 2C, 3A, 3B.

KRC: 2C, 3A, 3B.

## Disclosures

### Ethical Compliance Statement

Data used in the current analyses were obtained from the validation of the MDS‐NMS and the PKG Registry studies. For the MDS‐NMS study, each site contributing to the study received approval of their respective ethics committee/IRB for participation. In the UK, the study was approved for adoption to the UK Clinical Research Network (UKCRN No 18003). The PKG Registry obtained ethical approval from the London – Riverside Research Ethics committee (REC reference: 17/LO/1010). All patients gave written informed consent, and the study was conducted in line with the Declaration of Helsinki. We confirm that we have read the Journal's position on issues involved in ethical publication and affirm that this work is consistent with those guidelines.

### Funding Sources and Conflicts of Interest

The current data analyses were not supported by funding. There is no conflict of interest.

### Financial Disclosures for the Previous 12 Months

In the past year D.v.W. has received speaker fees from Bial and Britannia pharmaceuticals, and research grants from NIHR BRC and CHDI; S.R. has nothing to disclose; A.S. has nothing to disclose; A.R. has received support from the National institute of Health Research (NIHR) Clinical Research Network (CRN) South London, Guy's Hospital, Great Maze Pond, London UK and International Parkinson and Movement Disorder Society (MDS); P.M.M. has nothing to disclose; In the past year D.W. has received research funding or support from Michael J. Fox Foundation for Parkinson's Research, Alzheimer's Therapeutic Research Initiative (ATRI), Alzheimer's Disease Cooperative Study (ADCS), International Parkinson and Movement Disorder Society (IPMDS), National Institute on Health (NIH), Parkinson's Foundation; U.S. Department of Veterans Affairs and Acadia Pharmaceuticals; honoraria for consultancy from Acadia Pharmaceuticals, Alkahest, Aptinyx, Cerevel Therapeutics, CHDI Foundation, Clexio, Clintrex LLC (Otsuka), EcoR1 Capital, Eisai, Ferring, Gray Matter Technologies, Great Lake Neurotechnologies, Intra‐Cellular Therapies, Janssen, Merck, Sage, Scion, Signant Health and Vanda; and license fee payments from the University of Pennsylvania for the QUIP and QUIP‐RS. K.R.C. reports advisory board for AbbVie, UCB, GKC, Bial, Cynapsus, Lobsor, Stada, Zambon, Profile Pharma, Synovion, Roche, and Therevance, Scion, as well as honoraria for lectures for AbbVie, Britannia, UCB, Zambon, Novartis, Boeringer Ingelheim, Bial, Kyowa Kirin, SK Pharma, and frants (Investigator Initiated) from Bial, EU Horizon 2020, Parkinson's UK, NIHR, Parkinson's Foundation, Wellcome Trust, and royalties or licenses (ongoing) from Oxford (book), Cambridge publishers (book), MAPI institute (KPPS, PDSS 2), and payment for expert testimony for the General Medical Council (UK).
